# Risky Impulsive Behavior in Gilles de la Tourette Syndrome

**DOI:** 10.3390/jcm15187335

**Published:** 2026-09-21

**Authors:** Mateusz Grycz, Piotr Janik

**Affiliations:** 1 National Medical Institute of the Ministry of the Interior and Administration, 02-507 Warsaw, Poland; mateusz.grycz@pimmswia.gov.pl; 2 Department of Neurology, Medical University of Warsaw, 02-091 Warsaw, Poland

**Keywords:** risky impulsive behavior, impulsivity, Tourette syndrome, attention deficit hyperactivity disorder, oppositional defiant disorder

## Abstract

**Background/Objectives**: Behavioral disturbances are common in Gilles de la Tourette syndrome (GTS) and can include risky impulsive behaviors (RIBs), but RIB classification remains unclear. This study aims to define the epidemiology of RIB in GTS and to assess its relationship with tic severity and the prevalence of comorbid psychiatric disorders. **Methods**: The study was structured as a cross-sectional registration study, with each patient enrolled into the database during a single clinical visit. The study included 365 participants with GTS (272 males, 74.5%), of whom 278 (76.2%) were children and adolescents. The mean age at evaluation was 14.4 ± 9.8 years (range 4–64). Clinical data on RIB were obtained during routine outpatient visits using semi-structured questionnaires developed by the authors. **Results**: RIB was reported in 64 (17.6%) patients with GTS, a group composed of 19 (29.7%) adults and 45 (70.3%) minors. RIB commenced at a mean age of 8.1 ± 5.4 years (range 2–30). The mean age at onset of RIB was 2.6 ± 4.9 years after the onset of tics. Of the patients with RIB, 84.4% had at least one comorbid psychiatric disorder, whereas 15.6% of RIB patients had no psychiatric comorbidity. Multivariable logistic regression showed significant associations between RIB and YGTSS (*p* = 0.02, OR = 1.02), ADHD (*p* < 0.01, OR = 2.91), and ODD (*p* = 0.01, OR = 3.02). **Conclusions**: RIB may occur early in the course of GTS, typically beginning during childhood, and was identified in 17.6% of patients. Its occurrence was associated with the presence of comorbid psychiatric disorders, particularly ADHD and ODD. Greater tic severity was also statistically associated with RIB, although the effect was small and of limited clinical significance.

## 1. Introduction

Gilles de la Tourette syndrome (GTS) is a neurodevelopmental disorder characterized by the presence of both motor and vocal tics [1]. GTS typically begins in childhood and is frequently associated with behavioral disturbances and psychiatric comorbidities, including attention deficit hyperactivity disorder (ADHD), oppositional defiant disorder (ODD), self-harm behaviors (SHBs), and non-obscene, socially inappropriate behaviors (NOSIBs) [2,3,4]. In the International Classification of Diseases, 10th Revision (ICD-10), Tourette syndrome is classified under F95.2. During earlier clinical observations, we encountered behaviors in patients with GTS that did not align with the established categories of behavioral disturbance associated with the disorder. Examples included potentially risky actions or internal urges carrying a potential risk to oneself or others, such as running onto a busy street or riding a bicycle into pedestrians. Patients described these behaviors as impulsive and difficult to resist, while the actions were perceived as non-accidental and having a semi-purposeful character. It is important to note that the behaviours in question did not primarily constitute an attempt to cause bodily harm. The term “risky impulsive behaviour” (RIB) was thus employed to describe these potentially hazardous actions or internal urges.

RIB shares certain features with several behavioral phenomena encountered in GTS but also differs from them in clinically relevant ways. Risk-taking and poorly controlled behaviors are well recognized in ADHD and may result from deficits in response inhibition, planning, and delay of gratification [5]. However, ADHD-related impulsivity is typically described as part of a broader pattern of attentional and executive dysfunction, whereas RIB, as defined in the present study, was characterized by more discrete and abrupt behavioral episodes. Some patients with ODD may also display behaviors resembling RIB, while aggression and other harmful behaviors in conduct disorder (CD) may overlap phenomenologically with RIB. However, defiance, rule violation, and deliberate aggression are not defining features of RIB.

NOSIB may also resemble RIB because both behaviors can be intentional, impulsive, and difficult for patients to control [4,6,7,8]. The principal distinction is that NOSIB is characterized by social inappropriateness, whereas RIB is defined by the potential for physical harm and may occur during otherwise routine activities. SHB represents another important differential consideration. SHB generally involves deliberate bodily harm, with injury or the intention to cause injury being central to the behavior [3,9,10]. Conversely, RIB may result in injury, but physical harm is not its primary aim. Tic-related self-injurious behaviors may be inadvertent consequences of sudden, brief movements, whereas RIB consists of broader and more complex actions that do not resemble established tic phenomenology.

Stereotypic self-injury may similarly be distinguished from RIB by its repetitive, predictable, and often rhythmic nature. In contrast, RIB occurs in irregular and unpredictable episodes, which may be separated by days, weeks, or months. Another potentially overlapping phenomenon is stimulus-bound tics (SBTs), in which simple or complex tics occur in response to specific visual, auditory, or internal stimuli [11]. SBTs are generally brief, relatively simple, and rapidly executed, whereas RIB involves more prolonged and intricate actions and is not necessarily preceded by a specific stimulus [12].

The initial hypothesis was that RIB might represent a manifestation of coexisting psychopathology, particularly ADHD. However, during data collection, RIB was also identified in patients without psychiatric comorbidity and in adults seeking treatment for bothersome tics. These observations suggested that RIB might represent a distinct behavioral phenomenon that warrants investigation in its own right.

The present study used a large, consecutive sample of patients with GTS to investigate the prevalence, age of onset, and clinical characteristics of RIB. We specifically examined which psychiatric comorbidities are associated with RIB, whether RIB is associated with tic severity after adjustment for psychiatric comorbidities, and whether RIB occurs predominantly during childhood or also occurs among adults with GTS.

## 2. Materials and Methods

The study was structured as a cross-sectional registration study, with each patient enrolled into the database during a single clinical visit. The diagnosis of GTS, assessment of RIB, and classification into the RIB-positive and RIB-negative groups were performed during the same initial clinical evaluation visit. Follow-up data were not incorporated into the analysis. Ethical approval for the collection of clinical information from individuals diagnosed with GTS was granted by the Ethics Committee of the Medical University of Warsaw (KB/2/2007).

The present study subsequently included 365 consecutive patients with GTS seen between 2017 and 2024. The cohort of patients evaluated for the presence of RIB was identical to the one employed in our previous study [4]. From the initiation of the RIB study, all participants were assessed using the same semi-structured questionnaire and the same operational definition of RIB. Data from two patients were excluded because of missing information regarding RIB. Inclusion in the study was contingent upon meeting the established diagnostic criteria for GTS as outlined in the Diagnostic and Statistical Manual of Mental Disorders (DSM-5). A structured clinical interview was conducted using a questionnaire adapted from the Tourette Syndrome International Database Consortium Data Entry Form [13]. This tool was used to collect demographic details and screen for common comorbidities such as ADHD, ODD, obsessive–compulsive disorder (OCD), autism spectrum disorder (ASD), CD, depression, and anxiety disorders. Previous psychiatric diagnoses were accepted without re-evaluation at the time of assessment, as systematic verification would often have required retrospective diagnostic reconstruction. In many cases, relevant symptoms were no longer clinically present, making reliable reassessment difficult, particularly for conditions such as ADHD where symptoms may attenuate in adulthood. Re-evaluating past diagnoses based on historical symptom reports would have introduced a risk of recall bias and diagnostic misclassification. Therefore, previously established clinical diagnoses were used to minimise the limitations associated with retrospective verification in the absence of current symptomatology. Tic severity was assessed using the Yale Global Tic Severity Scale (YGTSS).

Following preliminary clinical observations made before the initiation of the present study, a semi-structured questionnaire was developed by the authors specifically to assess RIB. The questionnaire was not a previously validated instrument. The operational definition of RIB was established before assessment of the present study cohort and was developed to distinguish potentially hazardous, semi-purposeful behaviors that were difficult for the individual to resist from established tic phenomena, self-harm behaviors, socially inappropriate behaviors, and other psychiatric symptoms. The presence of the characteristic internal urge without enactment of the associated behavior was also considered within the RIB construct.

From the initiation of the present RIB study, all participants were assessed using the same semi-structured questionnaire and the same predefined operational definition of RIB. The assessment was based on information reported by the patient and/or parent and did not rely on direct observation of RIB during the clinical examination. Participants were classified as RIB-positive if at least one behavior (action or urge) met the predefined operational definition of RIB. Participants who did not report any of the qualifying behaviors were classified as RIB-negative. All examples listed in Appendix A of RIB-related behaviors were presented to patients and/or parents and read aloud sequentially by the same examiner, and responses were recorded in a yes/no format. Each participant was therefore presented with the same standardized examples to promote consistency in the initial assessment. To accurately distinguish between different disorders, face-to-face interviews were conducted. For younger children and individuals with severe ASD, parental reports were primarily used. Only after RIB had been identified was a more detailed assessment performed to characterize its features, including age of onset, current presence of the behavior, and its consequences. The current presence of RIB was assessed based on the patient’s or parent’s report of the behavior during the 1-month period preceding the assessment. Patients were also asked whether they actively avoided situations likely to trigger RIB or feared a recurrence of symptoms. Further inquiries addressed whether patients perceived their behaviors as symptomatic of a disorder (egodystonic) or as an intrinsic part of their personality (egosyntonic). Finally, patients were asked whether engaging in the RIB behavior provided them with a sense of relief (see Appendix A).

Statistical analyses were performed using RStudio for Windows version 2024.12.1+563 (© 2009–2025 Posit Software, PBC, Boston, MA, USA). Continuous variables were summarized using either the arithmetic mean and standard deviation or the median and interquartile range (IQR), as appropriate. Categorical variables were reported as absolute counts with corresponding percentages. For comparisons between two independent groups, the independent-samples *t*-test or the Mann–Whitney U test was used, as appropriate. The distribution of continuous variables was assessed using the Shapiro–Wilk test; variables showing significant deviation from normality were analyzed using the Mann–Whitney U test. For continuous variables analyzed using the Mann–Whitney U test, effect size was expressed as r, calculated as Z/√N, with 95% confidence intervals estimated using Fisher’s z transformation. For categorical variables, effect estimates were expressed as unadjusted odds ratios (ORs) with 95% confidence intervals. Given the exploratory nature of the study, univariate comparisons were performed without adjustment for multiple testing. Variables showing statistically significant associations (*p* < 0.05) in univariate analyses were subsequently considered for inclusion in the multivariable logistic regression model. Age and sex were included as covariates to account for potential confounding. These analyses were considered exploratory, and findings were interpreted accordingly.

## 3. Results

### 3.1. Prevalence of RIB

A total of 64 participants (17.6%) reported experiencing RIB at some point in their lives. The majority were male (*n* = 44, 68.8%), resulting in a male-to-female ratio of approximately 2:1 (Table 1). Among them, 19 out of 86 adults (22.1%) and 45 of 277 (16.2%) children and adolescents had a history of RIB.

### 3.2. Duration of RIB

Information on the duration of RIB was available for 53 of 64 subjects. At the time of assessment, 43 of 53 individuals (81.1%) continued to report RIB symptoms, while in 10 cases (18.9%) the behavior had resolved. Of those whose symptoms had ceased, two were adults, six were adolescents, and two were still children. The duration from RIB onset to evaluation spanned 0 to 12 years, with a mean of 3.8 years (SD = 3.4, median: 3).

### 3.3. Onset of RIB

Age-of-onset data were available for 43 of 64 individuals, showing a mean onset age of 8.1 years (SD = 5.4, range: 2–30 years), with a median of 7 years. RIB most commonly began in early childhood, with 30 cases (69.8%) manifesting by age 10. Among the five adults with available data on RIB onset, three (60%) reported onset before the age of 18 years. The subgroup for whom age of onset was unknown tended to be older, including 14 adults, 3 adolescents (aged 11–17) and 4 children. Data on both RIB onset and general tic onset were available for 42 participants. Within this subset, RIB typically emerged 2.6 years (SD = 4.9, median: 1, range: −5 to 23 years) after tic onset. Notably, 9 cases (21.4%) reported RIB before tics, and in 5 cases (11.9%), both began simultaneously.

### 3.4. Characteristics of RIB

Data regarding the effects of RIB on tension and its perceived intensity were gathered from 53 participants. An increase in tension following RIB was reported by 13 individuals (24.5%), while 5 (9.4%) experienced a reduction. Interestingly, one subject experienced either an increase or a reduction in tension depending on the type of RIB performed. For 18 participants (34.0%), RIB did not influence their sense of tension. The remaining 17 subjects (32.1%) could not determine whether RIB provided any relief.

### 3.5. Consequences, Suppression, and Awareness of RIB

Information on the consequences of RIB was available for 53 of 64 subjects. In 10 participants (18.9%), the characteristic internal urge was reported without subsequent enactment of the associated behavior. In 32 cases (60.4%), RIB was not associated with any observable consequences. Minor consequences were noted in 18 cases (34.0%), while major consequences requiring medical intervention occurred only in 3 cases (5.7%). Avoidance of situations likely to trigger RIB was reported by 10 participants (18.9%). Additionally, 17 individuals (32.1%) interpreted these inappropriate behaviors as a manifestation of illness, while 15 (28.3%) did not associate them with a disease. In total, 16 patients (30.2%) worried about the possibility of recurrence of symptoms, while 25 (47.2%) reported no such concern.

### 3.6. RIB Association with GTS Duration, Severity and Comorbid Psychiatric Disorders

The univariate analysis revealed significant associations between RIB and the duration of GTS, the severity of GTS as expressed by YGTSS, and the coexistence of ADHD, ODD, OCD, depression, and ASD (Table 1). The multivariate logistic regression analysis subsequently confirmed a significant association between RIB and YGTSS, as well as between the presence of ADHD and ODD (Table 2). A total of 30 patients (46.9%) with RIB had at least one coexistent externalising disorder, including ADHD, ODD, or CD, with 15 (23.4%) having at least two disorders. A total of 44 (68.8%) subjects were diagnosed with at least one co-occurring internalising disorder, including depression, anxiety, or OCD, with 22 (34.4%) having two or more such disorders. In total, 20 (31.3%) subjects had at least one concurrent diagnosis of both an internalising and an externalising disorder. Overall, 10 out of 64 (15.6%) RIB patients had no psychiatric comorbidity.

## 4. Discussion

To our knowledge, no previous studies have systematically assessed RIB as defined in the present study in patients with GTS. Thus, our findings provide an initial estimate of its frequency and an initial clinical characterization of its age at onset, relationship to tic onset, occurrence in adulthood, and associations with tic severity and psychiatric comorbidities. These findings should be considered preliminary and require confirmation using standardized definitions and validated assessment methods.

Our analysis demonstrated that 17.6% of GTS patients reported risky impulsive behaviors including actions and urges (RIBs). These behaviors typically occurred in middle childhood, with 7 out of 10 cases developing before the age of 10 years, and half before the age of 7 years. A noteworthy observation is the close temporal correlation between the onset of RIB and the age of tic onset, with approximately one-third of cases manifesting concurrently with, or even preceding, the emergence of tics. Despite this early onset, a delay of about four years was observed between RIB symptom onset and consultation in an expert clinic. Therefore, RIB may be an underrecognized phenomenon that often begins early in the course of GTS, as it may be misinterpreted as isolated misbehavior, part of another psychiatric disorder, rude behavior, or disregarded entirely.

Although GTS patients with comorbid RIB were slightly older at evaluation, disease duration did not emerge as a statistically significant variable in the multivariate analysis predicting RIB occurrence (Table 2). We hypothesize that reports of RIB onset in adolescence or adulthood are partly influenced by recall bias, as adults often attend visits independently and may have incomplete recollections of childhood symptoms. Moreover, RIB was identified in adult participants, indicating that it may also occur in adulthood. In our cohort, 22% of adults had RIB at the time of evaluation. Although both tics and ADHD symptoms may persist into adulthood [14,15,16], the presence of RIB in adults does not establish persistence of childhood-onset RIB. Among the five adults with available information on RIB onset, three (60%) reported that it had begun before adulthood. However, given the small number of adults with available onset data and the cross-sectional design, these findings cannot determine whether RIB represents persistence of childhood-onset behavior or onset later in life. In addition, in approximately one-third of RIB patients, the date of onset was not documented, again likely due to recall bias, given that the majority of this subgroup were adults.

RIB was reported twice as frequently in males compared to females, representing a lower male-to-female ratio than that observed for GTS in our cohort, where the ratio was 3:1. In the Polish adolescent population, externalising disorders, including ADHD and ODD, are reported to be more frequent in girls than in boys, with prevalence rates of 3.8% and 3.5%, respectively [17]. These two disorders were also the strongest correlates of RIB in our study. This may suggest that female sex represents a risk factor for RIB among patients with GTS and comorbid externalising disorders.

In our cohort, slightly more adults than children had a history of RIB. Although the onset is usually early, the behavior persisted at the time of evaluation in 4 out of 5 subjects. In the group where RIB had resolved before the assessment, remission typically occurred before adulthood. On the other hand, in our adult sample, 22% of adults reported RIB. Although we are aware that cross-sectional study does not allow us to conclude on the longitudinal course and symptom fluctuations in a particular patient, these findings may reflect the fluctuating course of RIB, which should be verified in further studies. The apparent “onset” of RIB in adulthood may therefore represent an intensification or recurrence of childhood symptoms, rather than a genuinely late onset. Given the observed association between RIB and tic severity, one possibility is that changes in tic severity over time may be accompanied by changes in RIB. However, this hypothesis remains speculative, given the cross-sectional design and the modest, clinically uncertain association between RIB and tic severity (r = 0.24, Table 1; OR = 1.02, Table 2).

Based on the occurrence of RIB in adults, clinicians should remain vigilant for unreported RIB in adult GTS patients, as such behaviors are embarrassing and socially unacceptable, and therefore rarely volunteered by the patients themselves.

Notably, approximately one-third of patients with RIB perceived RIB not as part of their illness, but as a habitual or characteristic behavior. Although RIB can theoretically lead to major physical injury, only 3 of 53 patients experienced serious consequences requiring medical attention. This may suggest that RIB is, at least in some cases, partially controllable, as 22% of patients reported being able to inhibit the behavior at the stage of an internal urge, and 19% avoided situations that could provoke it. In approximately 94% of cases, RIB resulted in minimal or no adverse consequences, suggesting that RIB is at least partially controllable in some patients, a phenomenon somewhat analogous to the suppression of tics in GTS. However, there is currently insufficient evidence to support this hypothesis conclusively.

The association between RIB and tic severity was statistically significant but of uncertain clinical significance. Together with the observation that RIB had little or no effect on internal tension in approximately two-thirds of patients, this finding is compatible with the hypothesis that RIB may differ phenomenologically from typical tics and may not be directly related to tic severity. However, this interpretation remains speculative and cannot be established by the present cross-sectional data. Furthermore, RIB often does not provide relief. Unlike tics, an increase in tension after RIB is three times more common than a reduction, likely due to the inherently dangerous and stressful nature of the acts for both patients and observers. We speculate that the nature of RIB and the ability to suppress it directly influences the effect on tension. For example, controlling RIB by stopping at the side of the road would reduce the feeling of tension, while running onto the road would increase the overall sensation of tension. This loss of inhibitory control could be correlated with more severe forms of RIB. The prefrontal inhibitory control circuit has been postulated to be associated with executive functioning and disinhibition and has also been linked to ADHD symptoms [18], which were associated with the presence of RIB in our study. The frontal and prefrontal cortex–striatum–thalamus–cortex circuit has also been documented to play a key role in tic and GTS pathophysiology [19]. RIB may therefore involve altered inhibitory control or executive functioning; however, our study did not include neurobiological or neuropsychological measures to test this hypothesis. These mechanisms require further investigation.

Approximately one-third of patients perceived RIB as a sign of a disease, 30% reported fear of recurrence, and one-fifth actively avoided situations that could trigger RIB. These findings indicate that RIB may be perceived as egodystonic by some patients, although perceptions varied considerably between individuals. It may be hypothesised that the nature of RIB and its severity affect the perception of the nature of the disorder by the patient. Notably, more severe and frequent behaviors may lead to active resistance, in a manner analogous to obsessive–compulsive phenomena observed in OCD. Consequently, such behaviors may be classified as egodystonic. Conversely, milder behaviors may be perceived as an integral component of an individual’s personality or temperament and therefore be regarded as egosyntonic, as was the case in approximately one-third of patients with RIB.

The results of our study indicate that patients with RIB manifested more severe tics and a higher prevalence of comorbid psychiatric disorders than patients without RIB (Table 1 and Table 2). In more severe cases of GTS, there is often a higher prevalence of comorbid psychopathology and inappropriate behaviors, such as RIB. RIB was strongly associated with comorbid ADHD and ODD, suggesting that it may be associated with disruptive behavior disorders. Tic severity appears to play a slightly lesser role in the occurrence of RIB than the coexistence of externalizing disorders (OR 2.91 and 3.02 for ADHD and ODD, respectively). Although greater tic severity was statistically associated with RIB, the multivariable effect per one-point increase in YGTSS was modest (OR 1.02, 95% CI 1.00–1.04). In the univariate group comparison, however, the difference in YGTSS corresponded to a small-to-moderate effect size (r = 0.24, OR 1.02). Thus, this statistically significant association should not be interpreted as evidence of a significant clinical effect. This suggests that the greater tic severity observed in patients with RIB may reflect a more complex psychopathological profile and an overall more severe clinical course, rather than a direct relationship between tic severity and RIB. From this perspective, the association between YGTSS scores and RIB may be secondary to co-occurring psychiatric disorders and greater overall clinical burden.

In the present study, RIB was found to be associated with internalising disorders, including depression and OCD, but not anxiety disorders. However, these associations were evident only in the univariate analysis, indicating a weaker relationship than that observed for ADHD and ODD. This finding suggests an overlap between externalising and internalising disorders within the study group, consistent with previous reports in young girls [20]. Notably, 15% of patients with RIB had no comorbid psychopathology, suggesting that, in this subgroup, tic severity may represent the primary risk factor. These findings indicate that clinicians should remain vigilant for RIB symptoms in all patients with GTS, regardless of the presence of coexisting psychiatric disorders.

Psychosocial interventions may be considered as a first-line approach for children with RIB, involving the child and relevant community members depending on the clinical and social context. Interventions should focus on increasing the child’s awareness of consequences, training parents to recognize and manage problematic behaviors, and implementing school-based strategies when needed. Comorbidities, particularly ADHD and ODD, should be identified and treated. Further research is warranted on the impact of tic-focused and comorbidity-targeted treatments on RIB. Recognition of RIB may help inform individualized behavioral and, when appropriate, pharmacological management in patients with GTS.

Taken together, our findings provide an initial clinical description of RIB in GTS rather than establishing it as a disorder-specific phenomenon. The observed frequency, early age at onset, occurrence in adults, and associations with ADHD, ODD and possibly tic severity identify potentially relevant clinical features that can be examined in future studies. Further research using validated assessment instruments and appropriate control or clinical comparison groups will be necessary to determine the specificity, clinical significance, and underlying mechanisms of RIB.

## 5. Limitations

The multivariable analysis was based on variables showing statistical significance in the univariate analyses. This data-driven approach may increase the risk of model instability and should therefore be considered exploratory.

A further limitation is that RIB was assessed using an author-developed, semi-structured questionnaire that was not formally validated. The questionnaire was developed following preliminary clinical observations made before initiation of the present study. Although standardized examples and face-to-face interviews were used, examiner judgment and differences in patient or parent reporting may have introduced classification bias, resulting in over- or underestimation of RIB. Therefore, the prevalence estimates and clinical associations should be interpreted cautiously, and validated assessment tools are needed.

Another important limitation is the absence of a control group. Because all participants had GTS, we could not determine whether RIB is more prevalent in GTS than in the general population or in individuals with other neurodevelopmental or psychiatric disorders. Consequently, the specificity of RIB for GTS and its distinction from behaviors occurring in other conditions cannot be established from the present study. Future studies including appropriately matched control groups and relevant clinical comparison groups are needed to determine whether RIB represents a behavior specifically associated with GTS or reflects a broader phenomenon related to impulsivity or psychopathology.

A further limitation concerns missing data for some RIB-specific characteristics. Missing data primarily affected the detailed characterization of RIB, as these questions were administered only to participants identified as RIB-positive. No formal missing-data mechanism analysis or imputation was performed; therefore, analyses of RIB-specific features were based on available cases, with the corresponding sample sizes reported in the manuscript. This approach may have introduced bias and limits the generalizability of findings concerning specific RIB characteristics.

The limitations of our study also include the inability to characterize the longitudinal course of RIB, including the timing of its resolution or whether childhood-onset RIB persists into adulthood. Although some participants reported that RIB was no longer present at the time of assessment, the cross-sectional design did not allow us to determine when or why the behavior had ceased. Longitudinal studies may provide further information on the natural history of RIB.

Additionally, we did not use a validated instrument specifically designed to evaluate the impact of pharmacological treatment or behavioral interventions on RIB, which limited our ability to assess treatment-related effects. Moreover, since participants were referred primarily to a neurology clinic, individuals with more severe behavioral disturbances or psychiatric comorbidities may have been more likely to be referred to psychiatric services instead. This referral bias may have contributed to an underestimation of RIB prevalence and associated psychopathology in our sample. Finally, the cross-sectional design limits the analyses to associations and precludes inference of causal relationships.

## 6. Conclusions

RIB may occur early in the course of GTS and may precede or coincide with tic onset in a subset of patients. Despite this, RIB is reported among adult patients more frequently than by minors. The clinical correlates of RIB include the presence of comorbid ADHD, ODD and possibly tic severity. RIB should be actively sought in all GTS patients, irrespective of patient age or the presence of comorbid psychopathology.

## 7. Take-Home Points

- RIB was identified in 17.6% of patients with GTS, suggesting that potentially hazardous behaviors may be relatively common but underrecognized in clinical practice.
- RIB usually began during childhood and often occurred close to the onset of tics, although it could precede tic onset and may occur in adulthood.
- RIB was particularly associated with comorbid ADHD and ODD. The correlation with tic severity was of uncertain clinical significance. These associations do not establish a causal relationship.
- RIB appears to be distinguishable from other behavioral problems such as ADHD-related impulsivity, self-harm, socially inappropriate behaviors, stereotypic behaviors, and stimulus-bound tics, although its diagnostic validity and specificity remain to be established.
- Clinicians should actively ask about RIB in patients with GTS, including adults and patients without psychiatric comorbidity, because these behaviors may be underreported and can potentially result in harm.
- Prospective studies using validated assessment tools and appropriate control groups are needed to establish the specificity, natural history, and clinical significance of RIB in the general population and other neurodevelopmental disorders.

## Figures and Tables

**Table 1:** Univariate comparisons between RIB present and RIB absent groups (*n* = 363, missing data were deleted casewise).

Variable	RIB Present (*n* = 64)	RIB Absent (*n* = 299)	Effect Estimate (95% CI)	*p*-Value
Gender (females), *n* (%)	20 (31.3)	73 (24.4%)	OR 1.41 (0.78–2.54)	0.331
Age at evaluation (years), median (IQR)	13.5 (9.0–20.5)	11.0 (9.0–16.5)	r 0.08 (−0.03–0.18)	0.095
**Disease duration (years), median (IQR)**	**5.5 (3.0–11.0)**	**5.0 (3.0–8.0)**	**r 0.12 (0.01–0.22)**	**0.032**
**YGTSS, median (IQR), min.–max.**	**56.5 (32.25–67.5), 8–98**	**38 (21.0–51.0), 7–88**	**r 0.24 (0.14–0.33)**	**<0.001**
**Depression, *n* (%)**	**19 (29.7)**	**28 (9.4)**	**OR 4.09 (2.11–7.93)**	**0.001**
Anxiety disorders, *n* (%)	35 (54.7)	122 (40.8)	OR 1.75 (1.02–3.02)	**0.233**
**ADHD, *n* (%)**	**25 (39.1)**	**36 (12.0)**	**OR 4.68 (2.54–8.63)**	**<0.001**
**OCD, *n* (%)**	**21 (32.8)**	**29 (9.7)**	**OR 4.55 (2.38–8.69)**	**<0.001**
**ODD, *n* (%)**	**19 (29.7)**	**23 (7.7)**	**OR 5.07 (2.56–10.05)**	**<0.001**
**ASD, *n* (%)**	**8 (12.5)**	**16 (5.4)**	**OR 2.53 (1.03–6.19)**	**0.039**

RIB, risky impulsive behavior; *n*, number; CI, confidence interval; IQR, interquartile range; r, effect size; YGTSS, Yale Global Tic Severity Scale; ADHD, attention deficit hyperactivity disorder; OCD, Obsessive–compulsive disorder; ODD, Oppositional defiant disorder; ASD, Autism spectrum disorder. Bold values are those statistically significant (*p* < 0.05). ORs shown in this table are unadjusted odds ratios.

**Table 2:** Multivariate logistic regression estimates (*n* = 363).

Variable	OR	95% CI	*p*-Value
Gender (females)	1.71	0.79–3.71	0.170
Age at evaluation	0.92	0.78–1.07	0.277
Disease duration	1.13	0.96–1.34	0.145
**YGTSS**	**1.02**	**1.00–1.04**	**0.015**
Depression	1.63	0.64–4.14	0.306
**ADHD**	**2.91**	**1.32–6.44**	**0.008**
OCD	2.10	0.92–4.80	0.078 *
**ODD**	**3.02**	**1.27–7.20**	**0.013**
ASD	1.86	0.64–5.37	0.251

*n*, number; OR, odds ratio; CI, confidence interval; YGTSS, Yale Global Tic Severity Scale; ADHD, attention deficit hyperactivity disorder; OCD, Obsessive–compulsive disorder; ODD, Oppositional defiant disorder; ASD, Autism spectrum disorder. Bold values are those statistically significant (*p* < 0.05). Values marked with * show a clear trend towards significance.

## Data Availability

The raw data supporting the conclusions of this article will be made available by the authors on request.

## Appendix A

Risky impulsive behaviors—examples:

- Risk to oneself:
- Has the urge to open a door or pull the handbrake while traveling in a car, deliberately closes their eyes while driving, recklessly turning the steering wheel while driving or while in the passenger seat,
- Has the urge to jump from a balcony,
- Stands close to the edge of a roadway/highway with the risk of being hit by a car,
- Runs onto a busy street,
- Leans dangerously out of a window or balcony railing with the risk of falling,
- Lets go of the handlebars while riding a bicycle with the risk of falling, rides a bicycle frantically in a public place or on a busy street,
- Leaves the house without telling their parents, risking becoming lost.
- Risk to others:
- Rides a bicycle into another person (pedestrian, another cyclist);
- Slams a door when another person walks through the door with the risk of causing injury to that individual’s hand, for example;
- Takes dangerous actions towards other people (e.g., pushes another person at the top of stairs).

Onset:

- Current: Yes/No.
- Tension: Reduction/Increase/No impact.

RIB intensity:

1. Urge without action;
2. Action started without completion;
3. Action completed;
4. Minor consequences of action;
5. Major consequences of action requiring medical intervention

Do you purposely avoid situations leading to the behavior? Yes—No—Don’t Know

Do you consider the behavior a sign of a disease? Yes—No—Don’t Know

Are you afraid that the behavior will occur again? Yes—No—Don’t Know
